# Kinesiophobia in stroke patients with type 2 diabetes mellitus: a cross-sectional study based on latent profile analysis

**DOI:** 10.3389/fneur.2026.1846279

**Published:** 2026-09-03

**Authors:** Ning Ban, Meng Guo, Susu Zhu, Xiao Miao, Daidi Tan, Liang Ma

**Affiliations:** 1 Neurosurgery Department, The Affiliated Lianyungang Hospital of Xuzhou Medical University, Lianyungang, Jiangsu, China; 2 Neurocritical Care Department, The Affiliated Lianyungang Hospital of Xuzhou Medical University, Lianyungang, Jiangsu, China; 3 Department of Critical Care Medicine, The Affiliated Lianyungang Hospital of Xuzhou Medical University, Lianyungang, Jiangsu, China; 4 Organization and Human Resources Department, The Affiliated Lianyungang Hospital of Xuzhou Medical University, Lianyungang, Jiangsu, China

**Keywords:** exercise self-efficacy, kinesiophobia, latent profile analysis, social support, stroke, type 2 diabetes mellitus

## Abstract

**Background:**

Exercise rehabilitation plays a critical role in promoting functional recovery after stroke. However, stroke patients with type 2 diabetes mellitus (T2DM) face additional challenges, including motor impairments, vascular lesions, and diabetic peripheral neuropathy, which may increase their susceptibility to kinesiophobia. Despite this, studies using latent profile analysis (LPA) to characterize kinesiophobia in this patient population remain limited. This study aimed to investigate the prevalence and latent subtypes of kinesiophobia among stroke patients with T2DM and to identify factors associated with each subtype.

**Methods:**

This cross-sectional study included 300 stroke patients with T2DM who were hospitalized in the Department of Neurology at a Grade 3 Class A hospital in Lianyungang City between August and December 2025. The Tampa Scale for Kinesiophobia was used as the primary assessment tool, and LPA was performed to identify distinct kinesiophobia subtypes. Univariate analysis and multinomial logistic regression were conducted to examine associations between kinesiophobia subtypes and sociodemographic, clinical, and psychosocial characteristics.

**Results:**

Three latent profiles of kinesiophobia were identified: a low-kinesiophobia group (*n =* 124, 41.3%), a medium-kinesiophobia group (*n* = 84, 28.0%), and a high-kinesiophobia group (*n* = 92, 30.7%). Multinomial logistic regression demonstrated that comorbidities, history of falls, duration of diabetes, Exercise Self-Efficacy (SEE), social support, pain, and fatigue were significantly associated with kinesiophobia subtype membership (all *p* < 0.05).

**Conclusion:**

Developing targeted interventions based on the subtype-specific features of kinesiophobia may help alleviate patients’ symptoms and promote their active participation in rehabilitation training. This is because three latent profiles of kinesiophobia were identified in stroke patients complicated with T2DM. In addition, the latent profiles exhibited differences in sociodemographic and clinical characteristics.

## 1 Introduction

Stroke is a chronic disease with a high global prevalence and has become a major public health challenge. It is the second leading cause of death worldwide, accounting for 11.6% of all deaths (1). Approximately 80% of stroke survivors experience varying degrees of functional impairment, particularly motor dysfunction, which substantially reduces quality of life (2). In China, the burden of stroke remains exceptionally high. Stroke is the leading cause of healthy life-year loss among the Chinese population (3) and is projected to remain the leading cause through 2050 (4). This substantial burden is closely associated with population aging and modifiable risk factors, including high-salt and high-fat diets, physical inactivity, hypertension, and dyslipidemia (5).

In addition to these general risk factors, metabolic comorbidities further increase the burden of stroke. T2DM, an independent risk factor for stroke, is strongly associated with stroke incidence and increases the risks of adverse post-stroke outcomes (6). T2DM is a leading metabolic risk factor for stroke, and the global stroke burden driven by hyperglycaemia and related metabolic abnormalities has increased over the past three decades (7). Compared with patients with stroke alone, those with stroke and T2DM experience not only sensory and motor impairments but also diabetes-related complications, such as peripheral neuropathy and retinopathy-associated visual impairment (8, 9). These complications further compromise motor function and balance, impede physical rehabilitation, and increase susceptibility to an excessive fear of movement, known as kinesiophobia (10, 11).

The Fear-Avoidance Model proposes that individuals experiencing disease-related physical discomfort exhibit two distinct behavioral responses. Continued participation in physical activity can gradually reduce fear of movement, whereas avoidance of activity reinforces catastrophic thinking. The latter response results in reduced physical activity and social participation, creating a vicious cycle of persistent pain, fear, impaired functioning, and progressive disability (12, 13). Approximately 78% of patients with stroke experience kinesiophobia (14). Kinesiophobia is strongly associated with greater activity-related pain, frailty, and an increased risk of falls (15). Consequently, it not only impedes rehabilitation among patients with stroke and T2DM but also adversely affects their physical and psychological well-being.

Despite its clinical significance, no standardized guidelines are currently available for the management of kinesiophobia, and many clinicians remain unfamiliar with evidence-based intervention strategies. This gap limits the effective implementation of clinical intervention. Furthermore, kinesiophobia reduces patients’ participation in rehabilitation, delays motor recovery, worsens clinical outcomes, and increases the burdens on families and healthcare systems (16). Therefore, the timely identification, assessment, and management of kinesiophobia are critical clinical priorities. Addressing this issue effectively is essential for promoting functional recovery and facilitating patients’ return to daily life.

However, managing kinesiophobia remains challenging, and no consensus has been reached regarding the optimal intervention strategy. Although recent systematic reviews have sought to optimize existing interventions for chronic conditions, treatment efficacy remains highly variable across individuals (17). These findings highlight that understanding patient heterogeneity is essential for developing targeted and effective intervention strategies. Existing research on kinesiophobia has primarily adopted a variable-centered approach to overall levels, with most studies focusing on populations with a single disease, such as chronic pain (18, 19), malignant tumors (20), or stroke alone (21). To date, few studies have specifically examined patients with stroke and comorbid T2DM or systematically investigated the heterogeneity of kinesiophobia using LPA.

The novelty of this study lies in shifting the analytical framework from the traditional variable-centered approach to a person-centered perspective. Using LPA, this study aimed to identify distinct kinesiophobia profiles among patients with stroke and T2DM. LPA identifies latent subgroups based on individuals’ observed responses and classifies those with similar characteristics into the same profile. Characterizing these profiles has important translational value and provides a foundation for developing stratified and individualized intervention strategies. This approach addresses the limitations of conventional one-size-fits-all rehabilitation programs and establishes a basis for the long-term and effective management of kinesiophobia in this comorbid population.

The present study aimed to (1) identify the latent profiles of kinesiophobia among patients with stroke and T2DM and (2) examine the factors associated with these distinct profiles. The findings provide a theoretical foundation for developing stratified and personalized intervention strategies.

## 2 Materials and methods

### 2.1 Participants

In this cross-sectional study, patients with stroke and T2DM admitted to the Department of Neurology at a Grade III Class A hospital in Lianyungang City were recruited using convenience sampling between August 28 and December 31, 2025. The inclusion criteria were as follows: (a) diagnosis of stroke confirmed by cranial computed tomography or magnetic resonance imaging (22); (b) diagnosis of T2DM according to established diagnostic criteria (23); (c) Montreal Cognitive Assessment score >26; (d) age ≥18 years; (e) stable vital signs; and (f) provision of informed consent. The exclusion criteria were as follows: (a) uncontrolled diabetes (fasting blood glucose ≥13.9 mmol/L); (b) serious visual, hearing, language, or cognitive impairment; (c) severe concurrent cardiac, hepatic, or renal dysfunction, or malignancy; (d) major life events within the preceding 3 months; and (e) withdrawal from the study or inability to cooperate with the study procedures.

### 2.2 Measures

#### 2.2.1 General information

The general information questionnaire was developed by the investigators based on a comprehensive literature review and expert consultation. The questionnaire collected participants’ basic sociodemographic characteristics.

#### 2.2.2 Kinesiophobia

The Tampa Scale for Kinesiophobia (TSK-17), developed by Miller et al. in 1991, was used to assess the severity of kinesiophobia (24). The scale comprises 17 items rated on a 4-point Likert scale. Items 4, 8, 12, and 16 are reverse scored. Total scores range from 17 to 68, with higher scores indicating greater kinesiophobia. A cutoff score of 37, which has been widely supported by previous studies and validated in Chinese patients with stroke (25), was used to identify participants with clinically significant kinesiophobia.

Regarding psychometric performance, Bąk et al. (26) reported that the TSK-17 demonstrated good reliability and validity in patients with stroke and T2DM. The scale showed an overall Cronbach’s *α* coefficient of 0.875, and confirmatory factor analysis supported a robust two-factor structure with acceptable model fit (CFI = 0.982, TLI = 0.979, RMSEA = 0.066, SRMR = 0.073). No significant floor or ceiling effects were observed for the total score, further supporting the construct validity of the TSK-17 in this comorbid population. The Chinese version of the TSK-17 was originally validated in individuals with chronic pain, and subsequent evidence has confirmed its suitability for assessing kinesiophobia in Chinese patients with stroke. In the present study, the scale demonstrated good internal consistency, with a Cronbach’s *α* coefficient of 0.855.

#### 2.2.3 Pain

Pain intensity over the preceding 24 h was assessed using the Numerical Rating Scale (NRS). The NRS uses an 11-point scale ranging from 0 to 10, where 0 indicates no pain, and 10 represents the worst possible pain (27). The NRS is a simple, easy-to-understand instrument suitable for patients of all ages and backgrounds and has been validated for use in Chinese patients with stroke (28). The Cronbach’s *α* coefficient for the NRS is 0.950.

#### 2.2.4 Self-efficacy for exercise (SEE)

The SEE Scale was developed by Resnick et al. in 2000 to evaluate individuals’ confidence in maintaining exercise despite barriers (29). The SEE consists of nine items, each scored from 0 to 10, yielding a total score ranging from 0 to 90. The Chinese version of the SEE has a Cronbach’s *α* coefficient of 0.750 (30). Confirmatory factor analysis has supported its construct validity (NFI = 0.90, RMSEA = 0.059). Furthermore, the SEE demonstrates satisfactory criterion and convergent validity, with significant positive correlations with objectively measured physical activity (*r* = 0.46) and exercise outcome expectations (*r* = 0.36) (30). Owing to its concise format and ability to capture common physical and psychological barriers to rehabilitation, the SEE has been widely used in studies involving Chinese patients with stroke (2). In the present study, the Cronbach’s *α* coefficient for the SEE was 0.957.

#### 2.2.5 Fatigue

Fatigue was assessed using the Neurological Fatigue Index for Stroke (NFI-Stroke). Developed by Mills et al. (31), the NFI-Stroke is a 12-item instrument that uses a 4-point Likert scale to assess fatigue in patients with stroke. Based on Rasch analysis of the original scale, items 8 and 10 are excluded from the total score calculation. Therefore, the total NFI-Stroke score is calculated by summing the scores for items 1–7, 9, and 11–12. The Chinese version of the NFI-Stroke has undergone localization and cultural adaptation and demonstrates high internal consistency, with a Cronbach’s *α* coefficient of 0.880. Comprehensive psychometric evaluation has also confirmed its strong measurement properties. The scale has excellent content validity (S-CVI = 0.950) and shows no significant floor or ceiling effects. Exploratory factor analysis identified a two-factor structure comprising lack of energy and tiredness/weakness, which adequately reflects fatigue symptoms in Chinese patients with stroke (32). In the present study, the Cronbach’s *α* coefficient for the NFI-Stroke is 0.936.

#### 2.2.6 Social support

Social support was assessed using the Social Support Rating Scale (SSRS), developed by Xiao Shuiyuan (33). Higher SSRS scores indicate greater perceived social support. The SSRS has demonstrated satisfactory validity and good internal consistency and has been widely used to assess social support among Chinese patients with stroke (34, 35). Previous validation studies have reported Cronbach’s *α* coefficients ranging from 0.880 to 0.940, supporting its reliability and validity. In the present study, the Cronbach’s *α* coefficient for the SSRS was 0.763.

### 2.3 Data collection

The research team established rigorous inclusion and exclusion criteria, and two uniformly trained investigators independently screened all patients. The investigators carefully documented the rationale for inclusion and the reasons for exclusion for each patient to minimize selection bias. This study was approved by the Ethics Committee (KY-20250828002-01). After providing written informed consent, patients completed the questionnaires independently in a quiet, distraction-free environment during hospitalization. Investigators provided only standardized clarifications for questionnaire items when requested.

Between August 28 and December 31, 2025, 330 patients were recruited for preliminary eligibility assessment at baseline. Four patients were excluded because of severe language impairment, and three were excluded due to severe concomitant organ dysfunction. Among the remaining 323 eligible patients, five declined participation. Ultimately, 318 patients provided written informed consent and received the study questionnaires. During questionnaire completion, seven patients withdrew because they were scheduled for diagnostic examinations, seven withdrew due to fatigue, and four withdrew because of acute changes in clinical condition. The final analytical sample consisted of 300 patients who completed all questionnaires. No significant differences in age or sex distributions were observed between patients who declined participation and those included in the final analysis.

**Figure 1:**
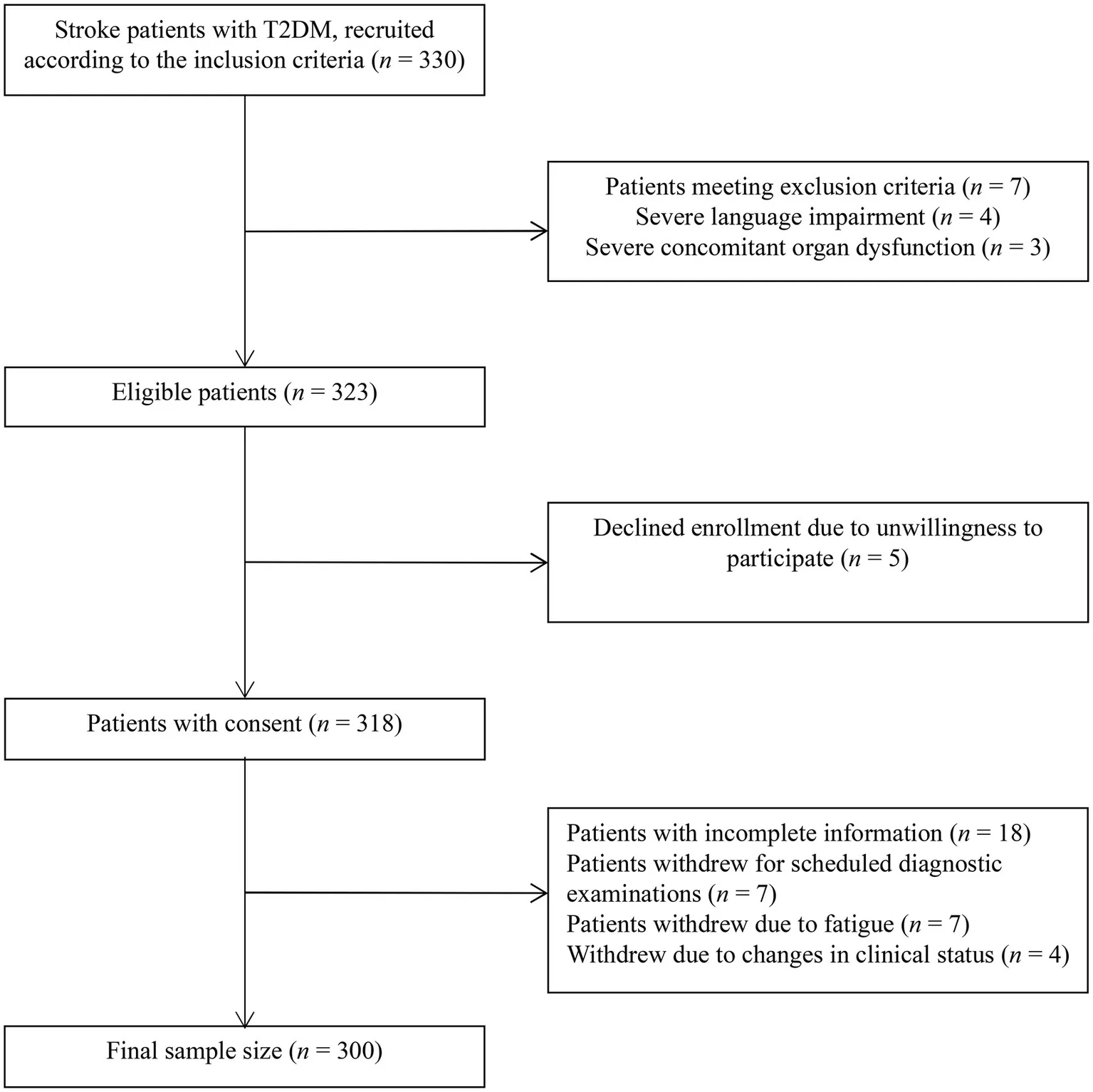
Flowchart of participant screening and recruitment. The diagram summarizes the number of patients at each stage, reasons for exclusion and withdrawal, and the final analytical sample of 300 patients.

Based on sample size requirements for LPA, each latent profile should include at least 50 participants. Therefore, a three-profile model required a minimum sample size of 150 participants. According to sample size estimation guidelines for cross-sectional studies, the required sample size should be at least 5–10 times the number of observed variables (36). This study included 23 observed variables. Assuming a 20% nonresponse rate, the estimated minimum sample size ranged from 128 to 288 participants. A total of 318 questionnaires were distributed, and 300 valid questionnaires were returned, resulting in a valid response rate of 94.34%. The sample size met the statistical requirements for LPA model development. The participant recruitment and data collection process is illustrated in Figure 1.

### 2.4 Statistical analysis

Data were analyzed using SPSS version 27.0 and Mplus version 8.3. Categorical variables were summarized as frequencies and percentages. Continuous variables were assessed for normality, and non-normally distributed variables were presented as medians and interquartile ranges (IQRs). Between-group comparisons were performed using the *χ*^2^ test or the Kruskal–Wallis *H* test, as appropriate.

LPA uses a model-based probabilistic framework that accounts for measurement error and provides robust statistical indices for identifying the optimal number of latent profiles. Therefore, it was selected over traditional distance-based clustering methods, such as K-means clustering. Moreover, LPA is specifically designed for continuous indicator variables, whereas latent class analysis is intended for categorical variables.

All latent profile models were estimated in Mplus version 8.3 using the robust maximum likelihood estimator, which is robust to non-normal data distributions. A one-profile model served as the baseline, and the number of latent profiles was increased sequentially. To reduce the likelihood of convergence to local maxima and ensure stable model estimation, 500 random initial starts and 100 final-stage optimizations were specified. Model stability was confirmed by successfully replicating the best log-likelihood value across repeated iterations.

Model fit was evaluated comprehensively using the Akaike Information Criterion (AIC), Bayesian Information Criterion (BIC), adjusted BIC (aBIC), entropy, Lo–Mendell–Rubin likelihood ratio test (LMRT), and bootstrap likelihood ratio test (BLRT). Lower AIC, BIC, and aBIC values indicate better model fit. An entropy value ≥0.8 indicates classification accuracy exceeding 90%. A statistically significant LMRT or BLRT result (*p* < 0.05) indicates that the k-profile model provides a significantly better fit than the (k–1)-profile model. In addition, each latent profile was required to include at least 5% of the total sample to avoid overstratification. The final profile solution was selected by jointly considering statistical performance, model parsimony, and the theoretical and clinical interpretability of the identified profiles.

## 3 Results

### 3.1 Participant characteristics

Among the 300 stroke patients with concomitant T2DM, 185 (61.67%) were male. The largest age group was 60–70 years (35.67%), and most patients had a junior secondary education or lower. A history of falls was reported by 79 patients (26.33%), and 139 patients (46.33%) had three or more comorbidities. Overall, 189 patients (63%) exhibited kinesiophobia. Detailed participant characteristics are presented in Table 1, and scale scores are shown in Supplementary Table 1.

**Table 1:** Participants’ sociodemographic and clinical characteristics (*n* = 300).

Variables	Number	Proportion (%)
Age (years)		
<60	68	22.7
60–70	107	35.6
71–80	98	32.7
>80	27	9.0
Gender		
Male	185	61.7
Female	115	38.3
Place of residence		
Rural	173	57.7
Urban	127	42.3
Level of education, *n* (%)		
Junior secondary school and below	230	76.7
Senior secondary school / Vocational school	43	14.3
College degree or above	27	9.0
Marital status		
Unmarried	4	1.3
Married	265	88.3
Widowed	26	8.7
Divorced	5	1.7
Monthly household income (RMB)		
<3,000	175	58.3
3,000–4,999	54	18.0
>5,000	71	23.7
Physical exercise		
No	79	26.3
1–2 times a week	105	35.0
≥3 times a week	116	38.7
BMI (kg/m^2^)		
<18.5	9	3.0
18.5–23.9	131	43.7
24.0–27.9	112	37.3
≥28.0	48	16.0
Economic burden of disease		
Lighter	57	19.0
Moderate	155	51.7
Heavier	88	29.3
Diabetes treatment		
Oral hypoglycaemic agents	55	18.3
Insulin injection	107	35.7
Oral and injectable medication	138	46.0
Comorbidities		
<3	161	53.7
≥3	139	46.3
Duration of diabetes (years)		
<20	196	65.3
≥20	104	34.7
Duration of stroke (days), *n* (%)		
1–7 days	270	90.0
8–14 days	27	9.0
≥15 days	3	1.0
History of falls		
No	196	65.3
Yes	104	34.7
TSK-17		
≤37 points	111	37.0
>37 points	189	63.0

RMB, Renminbi; BMI, Body Mass Index; TSK-17, Tampa Scale for Kinesiophobia.

### 3.2 LPA of kinesiophobia

Using the item scores of the TSK-17 scale as continuous indicator variables, an LPA model was developed for 300 stroke patients with T2DM. To identify the optimal model, one- to four- profile solutions (Models 1–4) were estimated sequentially. As the number of latent profiles increased, the AIC, BIC, and aBIC values generally decreased, consistent with the expected pattern of LPA model fitting. According to standard LPA criteria, an entropy value ≥0.8 indicates reliable classification, whereas a non-significant LMRT suggests that adding a latent profile does not significantly improve model fit. The three-profile model yielded higher entropy than the four-profile model (0.971 vs. 0.961). In addition, the four-profile model produced a non-significant LMRT result (*p* > 0.05), indicating that it did not provide a significantly better fit than the three-profile model. All latent profiles included at least 5% of the total sample, satisfying methodological requirements, and the three profiles demonstrated a clear gradient in kinesiophobia severity across clinical characteristics.

Overall, the three-profile model demonstrated lower AIC, BIC, and aBIC values, an entropy value >0.80, and high classification accuracy. Therefore, it provided the best balance between statistical performance, interpretability, and practical applicability and was selected as the optimal classification model for kinesiophobia in stroke patients with T2DM (Table 2).

**Table 2:** Parameters for the fitted latent profile model of kinesiophobia (*n* = 300).

Model type	AIC	BIC	aBIC	Entropy	*p*-value		Class probability
					LMRT	BLRT	
1	16990.27	17123.61	17009.44	—	—	—	—
2	15246.14	15449.85	15275.42	0.977	*p* < 0.001	*p* < 0.001	0.46/0.54
3	14723.54	14997.62	14762.94	0.971	*p* = 0.0008	*p* < 0.001	0.41/0.28/0.31
4	14400.60	14745.05	14450.11	0.961	*p* = 0.5451	*p* < 0.001	0.36/0.25/0.14/0.25

AIC, Akaike Information Criterion; BIC, Bayesian Information Criterion; aBIC, Adjusted Bayesian Information Criterion; LMRT, Lo–Mendell–Rubin Likelihood Ratio Test; BLRT, Bootstrap Likelihood Ratio Test.

To evaluate the reliability of the LPA classification, posterior probabilities of profile membership were calculated for each participant. The average classification probabilities for Profiles 1, 2, and 3 were 99.1, 97.8, and 99.3%, respectively, indicating excellent classification accuracy (Table 3).

**Table 3:** Probability of belonging to three latent profiles.

Latent profiles	C1	C2	C3
C1	0.991	0.009	0.001
C2	0.015	0.978	0.007
C3	0.000	0.007	0.993

C1, Low-kinesiophobia group; C2, Medium-kinesiophobia group; C3, High-kinesiophobia group.

**Figure 2:**
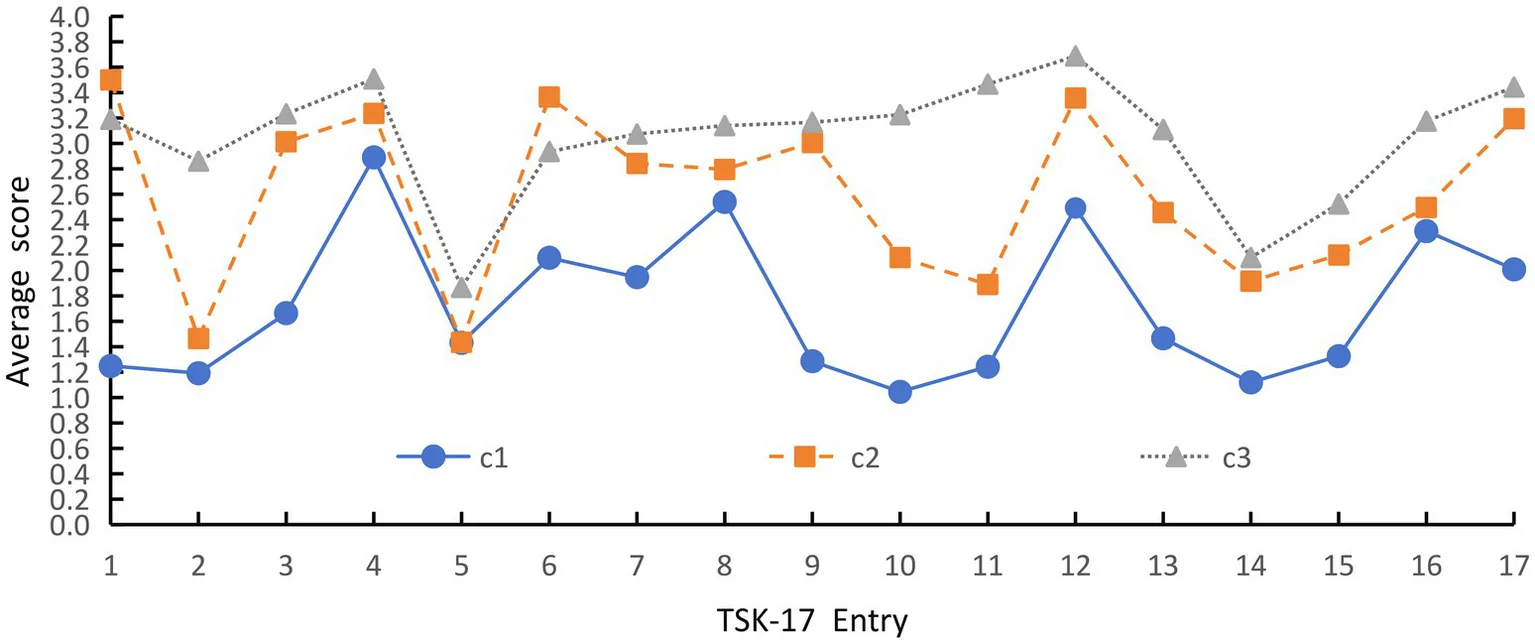
Latent profile characteristics of kinesiophobia in stroke patients with T2DM based on the 17 items of the TSK. The solid, dashed, and dotted lines represent the low-kinesiophobia (C1), medium-kinesiophobia (C2), and high-kinesiophobia (C3) groups, respectively. T2DM, type 2 diabetes mellitus; TSK-17, Tampa Scale for Kinesiophobia.

The three latent profiles included 124 patients in Categories 1 (C1; 41.3%), 84 patients in Category 2 (C2; 28.0%), and 92 patients in Category 3 (C3; 30.7%). The characteristics of the three latent profiles are illustrated in Figure 2.

Patients in C1 had a median total TSK-17 score of 30 (IQR, 29–35) and a mean item score of 1.73, indicating low kinesiophobia. These patients appeared able to regulate exercise-related fear effectively and demonstrated good adaptation to physical activity. Accordingly, C1 was classified as the low-kinesiophobia group. Patients in C2 had a median total TSK-17 score of 46 (IQR, 43–49) and a mean item score of 2.60. Compared with the other profiles, this group had higher mean scores for Item 1 (“I fear injuring myself if I exercise”) and Item 6 (“Accidents that occur will cause my body to remain at risk in the future”), indicating an exaggerated perception of exercise-related injury risk. This heightened fear of injury contributed to increased kinesiophobia and a more cautious attitude toward physical activity. Therefore, C2 was classified as the medium-kinesiophobia group. Patients in C3 had the highest kinesiophobia levels, with a median total TSK-17 score of 50 (IQR, 46–54) and a mean item score of 3.04. Compared with the other profiles, this group had higher scores for Item 4 (“If I exercise, the pain is likely to improve”), Item 12 (“I feel pain, but if I am active, the situation improves”) (both reverse-scored items), and Item 17 (“No one should exercise when in pain”). These findings suggest a negative cognitive bias regarding the relationship between exercise and pain, characterized by the belief that physical activity should be avoided when experiencing pain. Accordingly, C3 was classified as the high-kinesiophobia group.

### 3.3 Univariate analysis of factors associated with the latent profiles of kinesiophobia

Univariate analysis showed significant differences among the three latent profile groups with respect to sex, place of residence, educational attainment, monthly household income, perceived economic burden of illness, number of comorbidities, duration of diabetes, history of falls, NRS score, SEE scale score, NFI-Stroke score, and SSRS score (*p* < 0.05; Table 4).

**Table 4:** Univariate analysis of the latent profile of kinesiophobia (*n* = 300).

Variables	Number	C1 (*n =* 124)	C2 (*n* = 84)	C3 (*n* = 92)	*χ*^2^/*H*	*p* value
Age (years), *n* (%)						
<60	68	22 (17.7)	25 (29.8)	21 (22.8)	10.649	0.100
60–70	107	53 (42.7)	23 (27.4)	31 (33.7)		
71–80	98	35 (28.2)	32 (38.1)	31 (33.7)		
>80	27	14 (11.3)	4 (4.8)	9 (9.8)		
Gender, *n* (%)						
Male	185	86 (69.4)	52 (61.9)	47 (51.1)	7.459	0.024
Female	115	38 (30.6)	32 (38.1)	45 (48.9)		
Place of residence, *n* (%)						
Rural	173	63 (50.8)	60 (71.4)	50 (54.3)	9.322	0.009
Urban	127	61 (49.2)	24 (28.6)	42 (45.7)		
Level of education, *n* (%)						
Junior secondary school and below	230	84 (67.7)	72 (85.7)	74 (80.4)	14.258	0.007
Senior secondary school/Vocational school	43	28 (22.6)	4 (4.8)	11 (11.9)		
College degree or above	27	12 (9.7)	8 (9.5)	7 (7.6)		
Marital status, *n* (%)						
Unmarried	4	1 (0.8)	3 (3.6)	0 (0.0)	6.765	0.343
Married	265	111 (89.5)	73 (86.9)	81 (88.0)		
Widowed	26	11 (8.9)	7 (8.3)	8 (8.7)		
Divorced	5	1 (0.8)	1 (1.2)	3 (3.3)		
Monthly household income, *n* (%)						
<3,000	175	59 (47.6)	59 (70.2)	57 (61.9)	14.662	0.005
3,000–4,999	54	24 (19.4)	15 (17.9)	15 (16.3)		
>5,000	71	41 (33.1)	10 (11.9)	20 (21.7)		
Physical exercise, *n* (%)						
No	79	27 (21.8)	26 (31.0)	26 (28.3)	3.710	0.447
1–2 times a week	105	42 (33.9)	29 (34.5)	34 (36.9)		
≥3 times a week	116	55 (44.4)	29 (34.5)	32 (34.8)		
BMI, *n* (%)						
<18.5	9	2 (1.6)	5 (5.9)	2 (2.2)	5.780	0.448
18.5–23.9	131	60 (48.4)	31 (36.9)	40 (43.5)		
24.0–27.9	112	43 (34.7)	35 (41.7)	34 (36.9)		
≥28.0	48	19 (15.3)	13 (15.5)	16 (17.4)		
Economic burden of disease, *n* (%)						
Lighter	57	35 (28.2)	8 (9.5)	14 (15.2)	20.324	<0.001
Moderate	155	67 (54.0)	44 (52.4)	44 (47.8)		
Heavier	88	22 (17.7)	32 (38.1)	34 (36.9)		
Diabetes treatment, *n* (%)						
Oral hypoglycaemic agents	55	30 (24.2)	13 (15.5)	12 (13.0)	7.818	0.098
Insulin injection	107	41 (33.1)	36 (42.9)	30 (32.6)		
Oral and injectable medication	138	53 (42.7)	35 (41.7)	50 (54.3)		
Comorbidities, *n* (%)						
<3	161	92 (74.2)	39 (46.4)	30 (32.6)	39.189	<0.001
≥3	139	32 (25.8)	45 (53.6)	62 (67.4)		
Duration of diabetes (years), *n* (%)						
<20	196	103 (83.1)	53 (63.1)	40 (43.5)	36.800	<0.001
≥20	104	21 (16.9)	31 (36.9)	52 (56.5)		
Duration of stroke (days), *n* (%)						
1–7	270	115 (92.7)	72 (85.7)	83 (90.2)	8.745	0.068
8–14	27	9 (7.3)	9 (10.7)	9 (9.8)		
≥15	3	0 (0.0)	3 (3.6)	0 (0.0)		
History of falls, *n* (%)						
No	196	108 (87.1)	48 (57.1)	40 (43.5)	47.822	<0.001
Yes	104	16 (12.9)	36 (42.9)	52 (56.5)		
NRS, *M* (*P*_25_, *P_75_*)	—	0.0 (0.0, 0.0)	1.5 (1.0, 2.0)	2.0 (1.0, 3.0)	146.793	<0.001
SEE, *M* (*P*_25_, *P_75_*)	—	43.0 (27.0, 59.0)	39.0 (26.0, 49.0)	23.0 (9.0, 43.0)	28.502	<0.001
NFI-Stroke, *M* (*P*_25_, *P_75_*)	—	7.0 (0.0, 15.0)	13.0 (9.0, 19.0)	17.0 (12.0, 25.0)	53.908	<0.001
SSRS, *M* (*P*_25_, *P_75_*)	—	29.0 (25.0, 35.0)	25.0 (21.0, 31.0)	24.0 (17.0, 30.0)	33.104	<0.001

C1, Low-kinesiophobia group; C2, Medium-kinesiophobia group; C3, High-kinesiophobia group.

NRS, Numerical Rating Scale; SEE, Self-Efficacy for Exercise Scale; NFI-Stroke, Neurological Fatigue Index for Stroke, SSRS, Social Support Rating Scale.

### 3.4 Multinomial logistic regression analysis of the latent profiles of kinesiophobia

Multicollinearity diagnostics were performed before the multinomial logistic regression analysis. For the three core variables, SEE, NFI-Stroke, and SSRS, the variance inflation factors were 1.129, 1.277, and 1.261, respectively. All VIFs were well below the threshold of 2. In addition, pairwise Pearson’s correlation coefficients among the variables ranged from −0.203 to 0.151, all of which were substantially below the threshold of 0.7 for high correlation. All condition indices were <15. Collectively, these findings indicate no significant multicollinearity among these three variables; therefore, all were retained in the regression model.

With the low-kinesiophobia group (C1) as the reference group and incorporating univariate analysis results, the significant predictors were as follows: sex, place of residence, educational attainment, monthly household income, economic burden of illness, comorbidities, duration of diabetes, history of falls, NRS score, SEE score, NFI-Stroke, and SSRS. The variable coding methodology is detailed in Table 5.

**Table 5:** Assignment methods for independent variables.

Independent variables	Assignment methods
Gender	“Male” = 1; “Female” = 2
Place of residence	“Rural” = 1; “Urban” = 2
Level of education	“Junior secondary school and below” = 1; “Senior secondary school / Vocational school” = 2; “College and above” = 3
Monthly household income (RMB)	“<3,000” = 1; “3,000 ~ 4,999” = 2; “≥5,000” = 3
Economic burden of disease	“Lighter” = 1; “Moderate” = 2; “Heavier” = 3
Comorbidities	“< 3” = 1; “≥ 3” = 2
Duration of diabetes (years)	“< 20” = 1; “≥ 20” = 2
History of falls	“No” = 1; “Yes” = 2
NRS score	Original amount to be entered
SEE score	Original amount to be entered
NFI-Stroke score	Original amount to be entered
SSRS score	Original amount to be entered

NRS, Numerical Rating Scale; SEE, Self-Efficacy for Exercise Scale; NFI-Stroke, Neurological Fatigue Index for Stroke; SSRS, Social Support Rating Scale.

Multinomial logistic regression demonstrated that comorbidities, history of falls, diabetes duration, exercise self-efficacy, social support, pain, and fatigue were factors associated with kinesiophobia (*p* < 0.05). Compared with patients with ≥3 comorbidities, those with <3 comorbidities had lower odds of belonging to the medium-kinesiophobia group (OR = 0.369, 95% CI: 0.171–0.798) and high-kinesiophobia group (OR = 0.220, 95% CI: 0.089–0.542). Patients with diabetes duration <20 years had lower odds of belonging to the medium-kinesiophobia group (OR = 0.285, 95% CI: 0.119–0.679) and high-kinesiophobia group (OR = 0.151, 95% CI: 0.057–0.399). Patients with no history of falls had lower odds of belonging to the medium-kinesiophobia group (OR = 0.177, 95% CI: 0.073–0.433) and high-kinesiophobia group (OR = 0.097, 95% CI: 0.035–0.269). Patients with higher NRS scores were more likely to be classified into the medium-kinesiophobia group (OR = 2.663, 95% CI: 1.856–3.821) and high-kinesiophobia group (OR = 3.789, 95% CI: 2.535–5.664). Patients with higher NFI-Stroke scores were more likely to be classified into the medium-kinesiophobia group (OR = 1.050, 95% CI: 1.002–1.101) and high-kinesiophobia group (OR = 1.115, 95% CI: 1.055–1.179). Patients with higher SEE scores (OR = 0.962, 95% CI: 0.940–0.986) and SSRS scores (OR = 0.916, 95% CI: 0.855–0.982) were more likely to be classified into the low-kinesiophobia group (*p* < 0.05) (Table 6).

**Table 6:** Multinomial logistic regression analysis of the latent profile of kinesiophobia (*n* = 300).

Variables	B value	SE	Wald χ^2^	*p*-value	OR (95% CI)
C2 (vs. C1)					
Comorbidities (<3)	−0.997	0.394	6.422	0.011	0.369 (0.171, 0.798)
Duration of diabetes (<20 years)	−1.257	0.444	8.009	0.005	0.285 (0.119, 0.679)
History of falls (No)	−1.730	0.455	14.446	<0.001	0.177 (0.073, 0.433)
NRS score	0.979	0.184	28.247	<0.001	2.663 (1.856, 3.821)
NFI-stroke score	0.049	0.024	4.097	0.043	1.050 (1.002, 1.101)
C3 (vs. C1)					
Comorbidities (<3)	−1.515	0.460	10.837	0.001	0.220 (0.089, 0.542)
Duration of diabetes (<20 years)	−1.893	0.497	14.515	<0.001	0.151 (0.057, 0.399)
History of falls (No)	−2.328	0.517	20.272	<0.001	0.097 (0.035, 0.269)
NRS score	1.332	0.205	42.193	<0.001	3.789 (2.535, 5.664)
NFI-stroke score	0.109	0.028	14.763	<0.001	1.115 (1.055, 1.179)
SEE score	−0.038	0.012	9.754	0.002	0.962 (0.940, 0.986)
SSRS score	−0.088	0.035	6.193	0.013	0.916 (0.855, 0.982)

C1: Low-kinesiophobia group; C2: Medium-kinesiophobia group; C3: High-kinesiophobia group.

NRS, Numerical Rating Scale; SEE, Self-Efficacy for Exercise Scale; NFI-Stroke, Neurological Fatigue Index for Stroke; SSRS, Social Support Rating Scale.

## 4 Discussion

### 4.1 Kinesiophobia in stroke patients with T2DM exhibits group heterogeneity

Stroke patients with T2DM achieved a median TSK-17 score of 42 (IQR, 31–49), exceeding the threshold for kinesiophobia symptoms (37 points) and indicating a generally elevated level, which is consistent with a previous study (37). Unlike conventional aggregate-level research (38), in this study, LPA was used to identify three categories of kinesiophobia: groups C1, C2, and C3, which accounted for 41, 28, and 31% of the total study population, respectively. Notably, the combined proportion of groups C2 and C3 reached 59%, indicating that most stroke patients with T2DM present with elevated kinesiophobia levels. This finding further underscores the importance of identifying distinct categories of kinesiophobia. Clinically, precise patient stratification based on these categories provides a reference for clinical practitioners to implement tailored management strategies.

Within the low-kinesiophobia group, 48.4% of the patients had a BMI between 18.5 and 23.9 kg/m^2^. The proportions of marriage, secondary education or higher, urban residence, and lighter economic burden from illness were 89.5, 32.3, 49.2, and 28.2%, respectively. This group demonstrated relatively stable family circumstances and tended to have access to greater resources and support. Meanwhile, better overall health status and reduced disease-related economic burden among this population are positively associated with patients’ exercise self-efficacy. Such patients engage in regular physical activity voluntarily and strictly regulate blood glucose and body weight, which corresponds to lower kinesiophobia (39).

In the medium-kinesiophobia group, most patients have a monthly income <3,000 yuan (70.2%), a significant economic burden from illness (38.1%), and rural residence (71.4%). Furthermore, many patients had comorbidities, such as being overweight and a history of stroke. Excessive worries about discomfort or pain during physical activity are common among patients with insufficient rural medical resources, greater individual financial burden, and lower pain intensity. These worries are strongly associated with higher kinesiophobia (40).

Within the high-kinesiophobia group, the proportions of patients aged >70 years, those with no physical exercise, those with BMI ≥ 28.0, and those with diabetes duration ≥20 years were 43.5, 28, 17.4, and 56.5%, respectively. Furthermore, advanced age, physical inactivity, obesity, and long-standing diabetes are frequently accompanied by reduced sensation in the limbs and mobility limitations, which are associated with an increased risk of imbalance and falls during walking (41). Patients with such characteristics frequently hold the negative belief that exercise may cause injury. The correlations between these characteristics and kinesiophobia among stroke patients with T2DM are noteworthy.

Based on the three aforementioned profiles, we further illustrate targeted intervention strategies from the existing literature as reference examples for the stratified management of kinesiophobia. For the low-kinesiophobia group, leveraging patients’ initiative may be beneficial. Previous studies have suggested that exercise prescriptions can be effectively tailored to individual circumstances (42). For the medium-kinesiophobia group, health education focused on exercise safety awareness could be introduced to help address misconceptions. Concurrently, as suggested in previous studies, nonpharmacological pain management techniques might represent a potential strategy to alleviate exercise discomfort (43). For the high-kinesiophobia group, future clinical management should focus on identifying and gradually reshaping their negative perceptions of exercise (17). As documented in prior evidence, implementing such comprehensive strategies for stroke patients with T2DM may be considered in future clinical practice. These strategies may be associated with enhanced physical activity capacity and a reduction in associated risks (44).

### 4.2 Analysis of factors associated with the latent profile of kinesiophobia in stroke patients with T2DM

#### 4.2.1 Comorbidities

This study found that stroke patients with T2DM and three or more comorbidities were more likely to be classified into the medium- or high-kinesiophobia groups, consistent with the findings of Ozdemir et al. (45). One possible explanation is that patients with multiple comorbidities often experience impaired physical function and reduced mobility, which may be associated with greater concerns about exercise-related injuries, higher perceived organ burden, and stronger fears of aggravating existing medical conditions. These patients are more likely to exhibit pronounced kinesiophobia and avoidance behaviors during rehabilitation exercises. This finding highlights the need for greater clinical attention to stroke patients with T2DM who have three or more comorbidities. Based on existing literature, future clinical strategies may need to prioritize individualized risk assessment and comprehensive support to increase engagement in rehabilitation exercises (46).

#### 4.2.2 History of falls

This study showed that patients without a history of falls were more likely to be classified into the low-kinesiophobia group, consistent with previous findings (47). A possible explanation is that individuals without a history of falls generally have higher exercise self-efficacy, which is positively associated with intrinsic motivation to engage in physical activity (48). In contrast, a previous fall is strongly associated with more severe kinesiophobia. Clinically, patients with a history of falls often experience fear of reinjury accompanied by limitations in daily activities (49). Given this relationship, a history of falls may serve as an important early indicator for identifying patients at risk of kinesiophobia.

#### 4.2.3 Duration of diabetes

Our findings indicate that patients with a diabetes duration of <20 years are more likely to belong to the low-kinesiophobia group. Individuals with a shorter duration of diabetes generally experience less cumulative peripheral neuropathy and microvascular damage, which may contribute to better-preserved limb sensation, greater exercise tolerance, and a lower likelihood of physical discomfort (50). In clinical practice, stratified management based on diabetes duration may be beneficial. Existing evidence provides useful guidance for implementing such an approach. For patients with a shorter duration of diabetes and better exercise adaptability, reinforcing regular exercise guidance may help maintain physical function. In contrast, for patients with a longer duration of diabetes, comprehensive complication screening, individualized clinical management, and psychological interventions may be associated with better management of kinesiophobia.

#### 4.2.4 Exercise self-efficacy and social support

The present study found that patients with higher SEE scores and greater social support were more likely to be classified into the low-kinesiophobia group. This finding is consistent with previous research demonstrating the protective effects of exercise self-efficacy and social support on patients’ adaptation to exercise (51). A possible explanation is that patients with greater exercise self-efficacy and stronger social support are more likely to perceive professional guidance and family support positively. These favorable perceptions may reduce concerns about blood pressure and blood glucose fluctuations during physical activity. Concurrently, these patients tend to have greater confidence and a lower level of kinesiophobia when participating in rehabilitation exercises (52, 53). To address these factors in clinical practice, healthcare providers may adopt evidence-based strategies reported in the literature. For example, previous studies have shown that task-oriented training combined with immediate positive reinforcement after patients achieve incremental goals may be associated with greater confidence in exercise (54). In addition, establishing peer support groups is associated with better psychosocial well-being among stroke patients (55); such psychosocial improvements are linked to active engagement in physical rehabilitation.

#### 4.2.5 Pain and fatigue

This study found that stroke patients with T2DM who had higher NRS and NFI-Stroke scores were more likely to belong to the medium- and high-kinesiophobia groups. This finding is consistent with the Fear-Avoidance Model (12), which suggests that stressful events trigger defensive responses that are often manifested as avoidance behaviors associated with kinesiophobia. Previous studies have shown that both pain and fatigue are strongly associated with kinesiophobia, with each factor positively correlated with its severity (56, 57). Patients experiencing both pain and fatigue are more likely to adopt avoidance behaviors when facing rehabilitation challenges. The underlying reasons may be explained by two mechanisms (58). First, movement-related pain is often accompanied by negative perceptions of physical activity, which are closely linked to kinesiophobia. Second, persistent fatigue is commonly associated with slowed movement and a subjective sense of limb weakness.

To address these factors in clinical practice, several interventions reported in previous studies may help manage kinesiophobia. For instance, previous studies have indicated that outcomes among patients correlate favorably with deep breathing exercises, music therapy, or immersive virtual reality training (59, 60). For fatigue management, robot-assisted rehabilitation correlates with lower physical exertion and lower motor task complexity during training—factors linked to lower fatigue, which is often accompanied by lower kinesiophobia.

## 5 Limitations

This study has several potential limitations. First, as a single-center study with a relatively small sample size, the findings may have limited generalizability to other clinical settings. In addition, because the data were collected at a single time point, causal relationships among variables could not be established, and longitudinal changes in kinesiophobia could not be evaluated. Second, all study instruments were self-report questionnaires, making data collection dependent on patients’ subjective perceptions and response tendencies. Third, although LPA is effective for identifying population categories and offers advantages in revealing heterogeneity, the resulting classifications are influenced by model specification and selection, which may introduce subjectivity. Finally, this study did not include objective clinical indicators that may influence the development and severity of kinesiophobia, including hemoglobin A1c (HbA1c), diabetic complications (such as peripheral neuropathy or retinopathy), the National Institutes of Health Stroke Scale (NIHSS), and Barthel Index (BI), which may restrict our ability to fully evaluate their confounding roles. Additionally, the predominantly acute stroke study population(90.0% within 1–7 days) limited our ability to explore the impact of varying stroke durations. Despite these limitations, the findings may apply to patients with stroke and T2DM treated at tertiary hospitals in eastern China with similar demographic and clinical characteristics. Future large-scale, multicenter longitudinal studies across diverse countries and healthcare settings are warranted. Such studies should use long-term follow-up to characterize the dynamic trajectories of kinesiophobia across different latent profiles, establish causal relationships among variables, and further validate and extend the present findings.

## 6 Conclusion

Developing targeted interventions based on subtype-specific characteristics of kinesiophobia may help alleviate symptoms and encourage active participation in rehabilitation among patients with stroke and T2DM. In the present study, three distinct kinesiophobia subtypes were identified in this population. In addition, these subtypes differed in their sociodemographic and clinical characteristics. Recognizing these distinct profiles may enable healthcare professionals to implement tiered intervention strategies that address the heterogeneous needs of patients more effectively.

## Data Availability

The original contributions presented in the study are included in the article/Supplementary material, further inquiries can be directed to the corresponding author.
