# Supplementary material for: Kinesiophobia in stroke patients with type 2 diabetes mellitus: a cross-sectional study based on latent profile analysis
Source: Front Neurol. 2026 Sep 3;17:1846279. doi: 10.3389/fneur.2026.1846279 (PMC13581749; doi:10.3389/fneur.2026.1846279)
Supplement: Supplementary file 1 [file Table_1.pdf]

**Supplementary Table 1** Scale scores for stroke patients with T2DM (*n* = 300).

| Variables | Scores |
|---|---|
| TSK-17 | 42 (31–49) |
| NRS | 1 (0–2) |
| SEE | 38 (20–52) |
| NFI-Stroke | 13 (4–19) |
| SSRS | 26 (22–32) |

TSK-17: Tampa Scale for Kinesiophobia; NRS: Numerical Rating Scale; SEE: Self-Efficacy for Exercise Scale; NFI-Stroke: Neurological Fatigue Index for Stroke; SSRS: Social Support Rating Scale.
